# Radiation Exposure Related to Computed Tomography: Do We Need to be Unfazed, Scared or Very Scared?

**DOI:** 10.5334/jbr-btr.1034

**Published:** 2016-02-04

**Authors:** Nico Buls

**Affiliations:** 1 UZ Brussel, BE

## Short abstract to the handouts

In this presentation we will try to answer following questions: (1) what is the magnitude of medical radiation exposure compared to other EU countries, (2) how is medical exposure measured (especially in computed tomography (CT), (3) what are the involved stochastic health effects? A recent EU report (radiation protection no. 180, 2014) estimated the average effective dose (E) from medical exposure in Belgium to be around 2 mSv per year, which equals more or less the annual dose of natural background radiation. This is the highest value in the EU and can be explained by the high frequency of radiology procedures: with about 250 procedures per 1000 of population, Belgium has the highest frequency of procedures. Most EU countries are between 100 and 200 procedures per 1000 population. We learn that the assessment of effective dose from medical exposure—and especially CT—can be problematic due to the large heterogenic exposure geometry. We describe three methods to estimate organ and effective dose which have increasing level of complexity and cost: (1) generic Dose Length Product (DLP) to E coefficients, computer simulations with a (2) mathematical and a (3) detailed voxel model which is based on the actual patient images. Radiation effects from low doses (< 100 mSv) remain a debated issue. There are different models reported to extrapolate the effects which are observed at mediocre (between 100–2500 mSv) doses towards zero dose. The model which is currently the most defended is the linear no threshold (LNT) hypothesis. Population cancer risk estimates from medical exposures are typically based on these models. More recently, direct epidemiological studies are also reported on cancer risk in people exposed to CT in childhood or adolescence. However, we should acknowledge that our current knowledge on low dose effects remains inconclusive and we should avoid giving absolute estimates of cancer induction from medical exposure. Even patient dose estimations have many inherent limitations. Automated patient dose registration systems might help to improve accuracy in the future. Above all, the alara principle must prevail: doses should be kept as low as reasonably achievable by continuously optimizing scan protocols and by implementing new technological advantages.

## Competing Interests

The author declares that they have no competing interests.

